# Modifying dolutegravir to PrEPare for long life

**DOI:** 10.1038/s42003-022-04114-0

**Published:** 2022-11-07

**Authors:** Angelo Mandarino

**Affiliations:** Albert Einstein College of Medicine, Department of Microbiology and Immunology, The Bronx, NY 10461 USA

## Abstract

Anti-retroviral therapy with drugs like dolutegravir is a powerful tool in both the treatment and prevention of HIV, but is limited by strict adherence to a daily therapeutic regimen. In a recent study, Deodhar, Sillman, and colleagues developed a dolutegravir prodrug that offers long-lasting protection against HIV infection, with the potential to dramatically improve anti-retroviral therapy efficacy.

When HIV, the virus responsible for AIDS, was first identified in the early 1980s^1,2^, there were no treatment options or paths to recovery for patients. Four decades later, HIV infection has become a chronic, but manageable, condition with limited side effects or impact on patient life expectancy. Today, HIV infection can also be prevented by the use of some of the same anti-retroviral drugs (termed, pre-exposure prophylaxis, or PrEP) used to treat patients. Despite these advances, an effective HIV vaccine still remains elusive, meaning that the only option for treatment or prevention of HIV is anti-retroviral therapy with drugs like dolutegravir (DTG). However, anti-retroviral therapy demands strict adherence and daily drug dosing in order for it to succeed as a therapeutic or preventive agent, given the limited half-life of most therapeutic compounds.

In an effort to extend DTG half-life, Deodhar et al.^3^ used an ester bond to attach a lipophilic, 18-carbon fatty acid chain promoiety. This chemical modification significantly increases DTG’s half-life, improves drug transport, and decreases drug clearance rate, altogether extending DTG bioavailability from a single dose. The authors validated the prodrug’s longevity and bioavailability in several biological systems, ranging from in vitro monocyte-derived macrophages to in vivo rodent and macaque models. The prodrug was readily taken up by macrophages with limited cytotoxicity, and up to 30 days after treatment. Likewise, the prodrug had no toxic effect on mice, rats, or macaques up to a year after treatment, and had a reduced decay compared to unmodified DTG.

This work appears to offer one potential alternative to the strict dosing required by anti-retroviral therapy, and similar chemical modifications might eventually be applied to improve the stability of other therapeutic drugs. While the authors relied on in vitro or animal models, this prodrug could represent an important step towards improving HIV treatment, because long-lasting viral suppression can eventually lead to viral elimination.

**Figure:**
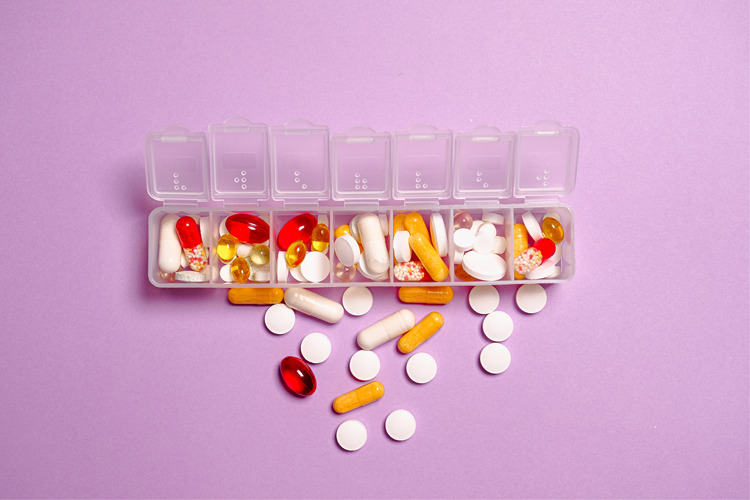
Anna Shvets
